# Is there an increased risk of cesarean section in obese women after induction of labor? A retrospective cohort study

**DOI:** 10.1371/journal.pone.0263685

**Published:** 2022-02-25

**Authors:** Jenny Bjorklund, Eva Wiberg-Itzel, Tove Wallstrom

**Affiliations:** 1 Department of Clinical Science and Education Karolinska Institute, Soderhospital, Stockholm, Sweden; 2 Womens Clinic, Soderhospital, Stockholm, Sweden; Lausanne University Hospital: Centre Hospitalier Universitaire Vaudois (CH), SWITZERLAND

## Abstract

**Background:**

Obesity is increasing in Sweden and is also of huge global concern. Obesity increases the risk of complications during pregnancy and the need for the induction of labor. Induction of labor increases the number of complications during delivery, leading to women with more negative birth experience. This study investigated how maternal body mass index (BMI) during antenatal care enrollment affects labor outcomes (proportion of cesarean section at induction of labor).

**Method:**

This was a retrospective cohort study of 3772 women with mixed parity and induction of labor at Soderhospital, Stockholm, in 2009–2010 and 2012–2013. The inclusion criteria were simplex, ≥34 gestational weeks, cephalic presentation and no previous cesarean section. The women were grouped according to BMI, and statistical analyzes were performed to compare the proportion of cesarean sections after induction of labor. The primary outcome was the proportion of cesarean section after induction of labor divided by group of maternal BMI. The secondary outcomes were postpartum hemorrhage >1000 ml, time of labor, fetal outcome data, and indication for emergency cesarean section.

**Result:**

The induction of labor in women with a high BMI resulted in a significantly increased risk of cesarean section, with 18.4–24.1% of deliveries, depending on the BMI group. This outcome persisted after adjustment in women with BMI 25–29.9 (aOR 1.4; 95% CI; 1.1–1.7) and BMI 30–34.9 (aOR 1.5; 95% CI; 1.1–2.1). There was also a significantly higher risk for CS among primiparous women (aOR 3.6; 95% CI; 2.9–45) and if the newborn weighted ≥ four kilos (aOR 1.6; 95% CI; 1.3–2.0).

**Conclusion:**

Our findings show that a higher BMI increased the risk of cesarean section after induction of labor in the groups with BMI 25–34.9. Parity seems to be the strongest risk factor for CS regardless other variables.

## Introduction

Overweight and obesity are increasing problems in Sweden, although they are also a huge global concern given the increased risk of complications they pose during pregnancy [1]. Studies have shown an increased risk of being overweight and obese at a lower socioeconomic status [2]. The proportion of Swedes with obesity has tripled since the 1980s [1]. In 2018, 26.4% of Swedish women were overweight (BMI>25), and 15.1% of them were obese (BMI>30) at enrollment in antenatal care. Large regional differences are present in the country; in some parts, half of all registered women are overweight or obese, while women in Stockholm have the lowest BMI in Sweden. Despite the lower incidence, as many as one in 4 women is overweight, and one in 10 women is obese in Stockholm [3].

Women with a high BMI have an increased risk of developing diabetes, preeclampsia (PE), and eclampsia during pregnancy [4, 5]. Maternal obesity is also associated with an increased risk for postdate pregnancy and fetal risks, such as large for gestational age (LGA), intra uterine growth restriction, intrauterine fetal death (IUFD), and Apgar score of <7 in 5 minutes [6–8].

Studies of gestational age in obese pregnancies have shown that a high maternal BMI increases the risk of a longer pregnancy (>41 weeks of gestation), which is associated with the risk of labor induction [8–11]. In the last five years, 21% of all pregnant women have been induced to labor in Sweden. Induction of labor (IOL) increase the risk of instrumental delivery, such as vacuum extractions and cesarean section (CS). The risk of CS after IOL was 22.6% vs. 9.4% in the spontaneous onset of labor in Sweden [12]. The corresponding figures for Soderhospital in Stockholm during the same period were 19.5% vs. 8% respectively [12]. Figures for obese women are even higher, where approximately 30% of obese women are delivered by emergency CS after a spontaneous onset of labor. Overweight and obesity are also associated with longer periods of active labor [14]. For cervical ripening and reaching active labor, studies have shown that obese women have a different course of labor compared with women with a normal weight (BMI <25) [7, 9, 10, 13, 14]. Cervical ripening is slower, which means that it takes longer to reach the active phase of labor and that they may need more doses of prostaglandins during IOL. Studies have also suggested that overweight and obese women may need higher doses of oxytocin during labor. Nevertheless, obese women have an increased risk of CS due to dystocia compared to normal-weighted women in normal deliveries, especially during IOL. In their assessment of the time of labor, Kobayash et al. showed [17] that the progress of labor differs between normal weights, overweight, and obese women. Obese women have slower progress between 4–7 cm of cervical dilation, and after that, the progress becomes normalized [15]. CS is more common in induced labor and is also associated with more serious complications. A previous CS increases the risk of a new CS, with an even higher risk for complications, especially in obese women. The aim of this study was to investigate how maternal body mass index (BMI) at antenatal care enrollment affects labor outcomes regarding the proportion of CS at IOL.

## Materials and methods

A retrospective cohort study of all induced women during the years 2009–2010 and 2012–2013 was conducted at Soderhospital, Stockholm, Sweden. The year of 2011 was not included because there was a change in clinical practice of IOL at the clinic in that year. One changed from Minprostin and balloon as primary method of IOL to oral solution of misoprostol. Since the method was new at the time, there were too many confounders present to use data from that particular year.

This hospital is the second largest maternity clinic in Sweden and a secondary referral center in the center of Stockholm, with nearly 8,000 deliveries a year. Women were grouped by BMI. Women with normal weight, G1 (= BMI <25), overweight, G2 (= BMI 25–29.9), and obesity were divided into two groups, G3 (= BMI 30–34.9) and G4 (= BMI >35).

The primary outcome was the proportion of CS after IOL divided by group of maternal BMI. Secondary outcomes were postpartum hemorrhage (PPH) >1000 ml, time of labor (from induction to delivery), indication for emergency CS, Apgar score <7 at 5 min, and cord blood pH <7.1. The inclusion criteria were induced women with simplex pregnancy ≥34 weeks, cephalic presentation and no previous CS. The exclusion criteria were multiple births, non-cephalic position, intrauterine fetal death (IUFD), and known fetal malformation or pregnancy <34 weeks of gestation. As the incidence of women with a previous CS is very low (only 5%) in this study, they were removed from the study to give an even more homogeneous group with women with no history of previous CS.

All personal data were encoded so that individuals could not be identified in the analysis.

### Statistics

Data were collected from the journal system Obstetrix (Cerner, *North Kansas*). Statistical analyses were performed using SPSS (23.0) (*SPSS Inc*., *Chicago*, *Illinois*). Categorical variables are presented as frequencies (%), and continuous variables are presented as mean values (with standard deviation (SD)). The method for comparing the means of continuous variables was an independent sample t-test used. To compare binary variables, calculations were performed either by Fisher’s exact test in groups <5 or Chi-square test for the others.

Binary logistic regression was used to study the risk of CS after IOL. Associated factors examined were BMI group (G1–4), age (<30 or >30 years), previous vaginal delivery (yes/no), gestational age (>41+0v or <41+0v), Bishop score (BS) at the start of induction (>5 or <5), indication for IOL, oxytocin infusion (yes/no), systolic blood pressure >140mmHg (yes/no), and PPH >1000 ml during childbirth. Fetal birthweight (>4000 g or <4000 g). The crude (unadjusted) association was first calculated for each possible explanatory variable. Variables that showed significant association were analyzed with multivariable models to study the adjusted associations with respect to the other possible explanatory variables (above). The associations are presented as odds ratios (OR) with a 95% confidence interval (CI). A p-value <0.05 was considered statistically significant.

### Ethical approval

The study was approved by the regional ethics committee (Karolinska Institute, file record: 2014/757-31/2). The study complies with the World Medical Association Helsinki Declaration regarding the ethical conduct of research involving human subjects.

## Results

**Fig 1:**
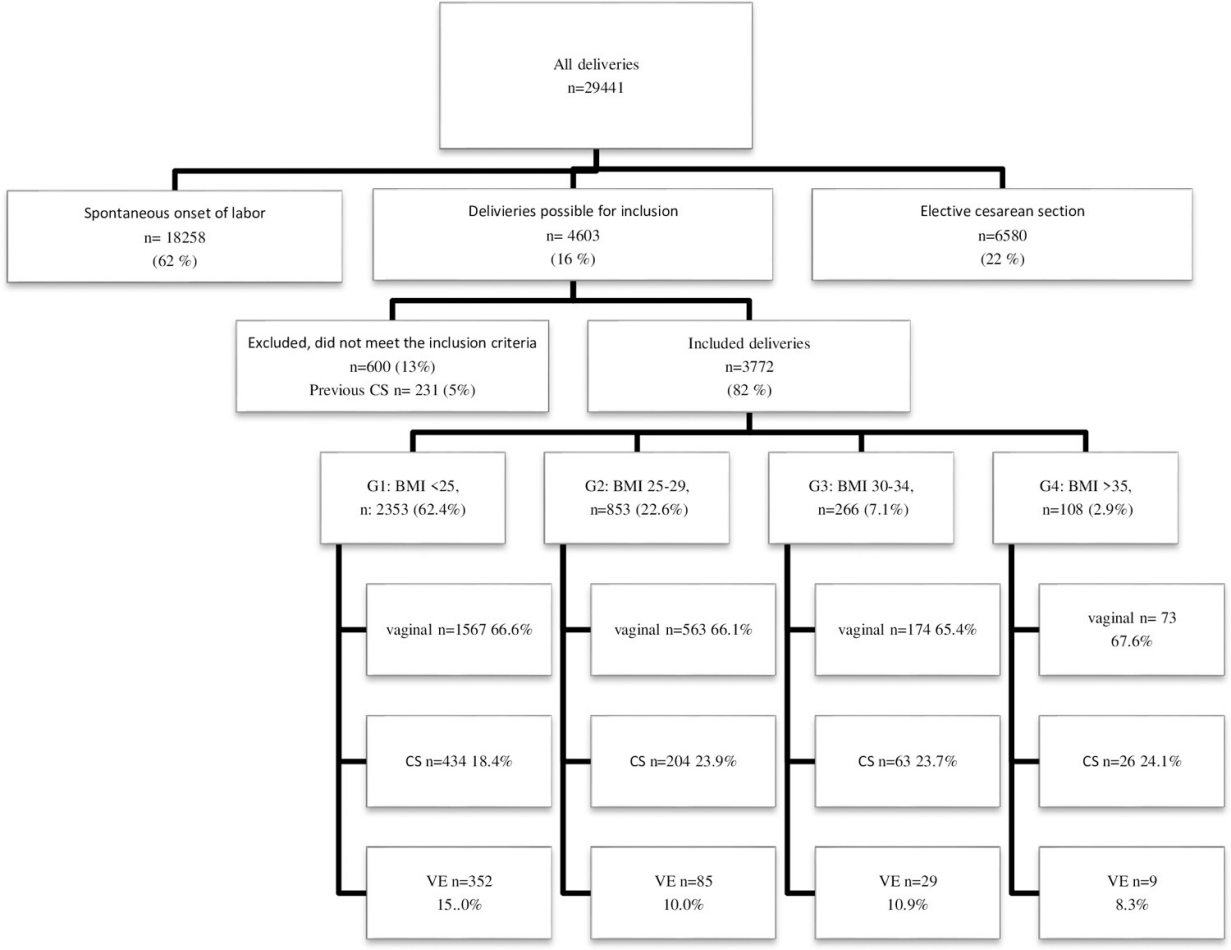


During the study time, 29441 women were delivered at the hospital. Of these, 4603 (16%) of the women were induced to labor (Fig 1) and 3772 of these induced women met the inclusion criteria for this study and constituted the study sample. The proportion of overweight women (BMI 25–29.9) in the study was 22.6% (853/3772), 7.1% (266/3772) of them were obese (BMI ≥30–34.9) and 2.9% (108/3772) had severe obesity (BMI >35). This is consistent with the pregnant population in Stockholm. Maternal baseline data showed significant difference (33.1–33.9 years, p = 0.003) between the BMI groups (G1–G4) according to maternal age, gestational age. There were no significant difference in Bishop score (BS), gestational age, or method of induction between the four groups (Table 1).

**Table 1:** Maternal baseline data. Women who underwent induction of labor (IOL) (n = 3772) grouped according to BMI (G1-G4). Data are presented as numbers (%) or mean (standard deviation [SD]). P-values <0.05 were considered statistically significant*.

	*G1 BMI (<25)*	*G2 BMI (25–29*.*9)*	*G3 BMI (30–34*.*9)*	*G4 BMI (≥35)*	*p-value*
	*n = 2353*	*n = 853*	*n = 266*	*n = 108*	
Maternal age (years)	33.1 [4.9]	33.1 [5.0]	33.5 [5.9]	33.9 [5.2]	0.03*
Parity					
Primiparous (n)	1522 (64.7%)	515(60.4%)	143 (53.8%)	57 (52.8%)	<001*
Indications for IOL (n)					<0.01*
PROM##	528 (22.4%)	191 (22.4%)	48 (18.0%)	17 (15.7%)	
Postdate pregnancies	669 (28.4%)	229 (26.8%)	61 (22.9%)	17 (15.7%)	
Maternal**	386 (16.4%)	152 (17.8%)	66 (24.8%)	39 (36.1%)	
Fetal^^	270 (11.5%)	94 (11.0%)	19 (7.1%)	13 (12.0%)	
Non-medical ***	500 (21.2%)	187 (21.9%)	72 (27.1%)	22(20.4%)	
Gestational age (days)	283 [12]	282 [12]	282 [12]	280 [12]	0.19
BS^ (n)					
<5	1645 (70.2%)	607 (71.2%)	196 (73.7%)	76 (70.4%)	0.73
≥5	709 (29.8%)	246 (28.8%)	70 (26.3%)	32 (29.6%)	
BP*(mm Hg)	123/ 79	125/81	129/83	133/83	<0.01*
Oxytocin infusion (n)	1491 (63.3%)	547 (64.1%)	159 (59.8%)	67 (62.0%)	<0.01*
Method for IOL					0.30
Cytotec	977 (41.5%)	385 (45.1%)	115 (43.2%)	43 (39.8%)	
Minprostin	535 (22.7%)	195 (22.9%)	69 (25.9%)	30 (27.8%)	
Balloon catheter	204 (8.7%)	61 (7.2%)	17 (6.4%)	4 (3.7%)	
Amniotomy	456 (19.4%)	154 (18.1%)	45 (16.9%)	26 (24.1%)	
Oxytocin infusion	134 (5.7%)	49 (5.7%)	16 (6.0%)	3 (2.8%)	
Propess	47 (2.0%)	9 (1.1%)	4 (1.5%)	2 (1.9%)	

#CS-cesarean section

##Pre labor rupture of membranes—PROM

*Blood pressure-BP

^Bishop score—BS, Maternal

**—elevated blood pressure, PE, pain, other disease, Fetal

^^-intrauterine growth restriction/LGA, blood flow impact, decreased fetal movements

*** Non-medical reason, woman request.

There was a significant difference according to parity, where nulliparous women were more common in group G1. It was significantly more women that needed oxytocin infusion in group G2 (64.1%) compared to G3 that the lowest figures (59.8, p<0.001). Significant difference was also found in the indication for IOL (p <0.01). Postdated pregnancies were the most common indication in Groups G1 and G2, non-medical indication was the most common in G3, and maternal indication was the most common in G4. No significant difference in IOL method was found between the groups (p = 0.8). Differences in blood pressure at admission to the delivery ward were significant; however, they were normal in all four groups (p <0.01).We found that 19.3% (727/3772) of IOL ended in a CS. The highest frequency of CS was found in G4 compared to the group with the lowest BMI in G1 (24.1% vs. 18.4%, p <0.01, Table 2).

**Table 2:** Birth outcome presented by BMI group (G1-G4). Data are presented as numbers (%) or mean (standard deviation [SD]). P-values <0.05 were considered statistically significant*.

	G1 BMI <25	G2 BMI 25–29.9	G3 BMI 30–34.9	G4 BMI ≥35	p-value
	n = 2353	n = 853	n = 266	n = 108	
Mode of delivery (n)					
Non instrumental	1567 (66.6%)	563 (66.1%)	174 (65.4%)	73 (67.6%)	<0.01*
vacuum extraction	352 (15.0%)	85 (10.0%)	29 (10.9%)	9 (8.3%)	
esarean section	434 (18.4%)	204 (23.9%)	63 (23.7%)	26 (24.1%)	
PPH (n)					
>1000 ml	107 (4.9%)	46 (5.9%)	13 (5.4%)	6 (6.5%)	0.92
Time from induction to delivery (h)	16.5 [10.8]	17.0 [11.2]	18.1 [11.5]	18.0 [11.8]	0.06
Indication for CS^(n)					
Primary dystocia	136 (31.9%)	82 (36.0%)	30 (43.5%)	14 (38.9%)	0.6
Secondary dystocia	60 (14.1%)	25 (11.0%)	8 (11.6%)	2 (5.6%)	
Threatening fetal asphyxia	191 (44.8%)	91 (39.9%)	25 (36.2%)	17 (47.2%)	
Fetal position	20 (4.7%)	11 (4.8%)	4 (5.8%)	0 (0%)	
Maternal**	11 (2.6%)	9 (3.9%)	2 (2.9%)	1 (2.8%)	
Ablatio	1 (0.2%)	0 (0%)	0 (0%)	0 (0%)	
Other	7 (1.6%)	7 (3.5%)	0 (0%)	1 (3.8%)	

^cesarean section -CS, Maternal

**—exhausted mother, pain, other disease.

Vacuum extraction (VE) was significantly more common with vacuum among women with normal BMI compared to the overweight women (15.0% vs. 8.3–10.9%, Table 2). There was no significant difference in the frequency of PPH >1000ml, time from induction to delivery, or indication for emergency CS between the four groups (Table 2).

There was a significant difference in fetal weight (p <0.01). Fetal weight increased with maternal BMI (Table 3), and 21.4% (806/3772) of the newborns had a fetal weight ≥4000g. No significant difference was seen regarding Apgar <7 at 5 min or cord blood pH <7.10 (Table 3).

**Table 3:** Fetal outcome presented by BMI group (G1-G4). Data are presented as numbers (%) or mean (standard deviation [SD]). P-values <0.05 were considered statistically significant*.

	G1 BMI <25	G2 BMI 25–29.9	G3 BMI 30–34.9	G4 BMI ≥35	p-value
	n = 2353	n = 853	n = 266	n = 108	
Fetal weight(g)	3549 [558]	3664 [544]	3719 [567]	3693 [591]	<0.01*
Fetal weight (n) ≥4000g	466 (19.8%)	233 (27.3%)	76 (28.6%)	31 (28.7%)	<0.01*
Fetal length (cm)	50.8 [2.4]	51.0 [2.2]	51.1 [2.4]	51.1 [2.2]	0.11
Apgar <7 at 5’(n)	18 (0.8%)	3 (0.4%)	2 (0.8%)	2 (1.9%)	0.29
pH <7.10** (n)	78 (3.3%)	21 (2.5%)	11 (4.1%)	4 (3.7%)	0.49

**Missing: n = 477.

The associations between the different risk factors for CS and IOL are presented in Table 4. A significant difference was seen regarding the risk that the IOL ended in a CS in groups G2 (aOR 1.4; 95% CI 1.2–1.7) and G3 (aOR 1.; 95% CI 1.1–1.9), but not significant in G4. After adjusting for other risk factors, the difference was still significant in G2 and G3 (aOR 1.4; 95% CI; 1.1–1.7 and aOR 1.5; 95% CI; 1.1–2.1, Table 4).

**Table 4:** Association between potential risk factors for cesarean section (CS) in women who underwent induction of labor (IOL). Data are presented as numbers (%) or mean (standard deviation [SD]). P-values <0.05 were considered statistically significant*.

Risk factors for CS after IOL	CS/total (%)	OR unadjusted (95%CI)	OR adjusted (95% CI)
BMI (n)			
G1 ≤24.9	434/2353 (17.1)	Ref	
G2 25–29.9	204/853 (23.9)	1.4 (1.2–1.7) *	1.4 (1.1–1.7) *
G3 30–34.9	63/266 (23.7)	1.4 (1.1–1.9) *	1.5 (1.1–2.1)*
G4 ≥35	26/108 (24.0)	1.4 (0.9–2.2)	1.3 (0.8–2.2)
Maternal age (years)			
<30	155/844 (18.4)	Ref.	
≥30	613/2927 (20.9)	1.2 (1.0–1.4)	1.5 (1.2–1.8) *
Parity (n)			
Multiparous	143/1424 (10.0)	Ref.	
Primiparous	625/2347 (26.6)	2.3 (1.9–2.7) *	3.6 (2.9–4.5) *
Gestational age (weeks)			
<41+0	321/2000 (16.1)	Ref.	
≥41+0	447/1770 (25.3)	1.8 (1.5–2.1) *	1.6 (1.3–2.0) *
BS^ (n)			
>5	164/1109 (14.8)	Ref.	
≤5	604/2662 (22.7)	1.7 (1.4–2.0) *	1.6 (1.3–2.0) *
Indication for IOL (n)			
PROM	133/829 (16.0)	Ref	
Postdate	242/1026 (23.6)	1.6 (1.3–2.0) *	1.0 (0.7–1.4)
Maternal**	185/679 (27.2)	2.0 (1.5–2.5) *	1.8 (1.3–2.4) *
Fetal^^	82/414 (19.8)	1.3 (1.0–1.7)	1.3 (0.9–2.1)
Non-medical	126/823 (15.3)	1.0 (0.7–1.2)	1.3 (0.9–1.9)
Fetal weight (n)			
<4000 g	536/2922 (18.3)	Ref	
>4000 g	232/849 (27.3)	1.7 (1.4–2.0) *	1.6 (1.3–2.0) *
Oxytocin infusion (n)			
No	298/1507 (19.8)	Ref.	
Yes	470/2264 (20.8)	1.1 (0.9–1.3)	1.0 (0.8–1.4)
Systolic BP* (mm Hg)			
<140	507/2648 (19.1)	Ref.	
>140	195/709 (27.5)	1.6 (1.3–1.9) *	1.4 (1.1–1.7) *

BS^- Bishop score, BP*—blood pressure, Maternal**- elevated blood pressure, PE, pain, other disease, Fetal^^- intrauterine growth restriction/LGA, blood flow impact, decreased fetal movements.

After adjustment for other risk factors, parity seemed to be strongest risk factor for CS. Further, the analysis showed an increased risk of CS after IOL if the woman was ≥30 years old, nulliparous, BS ≤5, or was induced ≥41+0 weeks. The results also showed that the risk of CS was increased with IOL due to maternal indication (aOR 1.4; CI 95% 1.0–2.0), fetal weight >4000 g (aOR 1.8; 95% CI 1.4–2.2), or a woman’s systolic blood pressure was >140 mm Hg (aOR 1.6; 95% CI 1.2–2.0) upon presenting for delivery. However, oxytocin infusion did not increase the risk of CS Table 4). Interactions were found between maternal age and parity p-value <0.001 and between parity and indication for IOL, p-value 0.02. There were no further interactions (p-values between 0.06–1.0).

## Discussion

The main finding of the study was the increased risk of CS after IOL with a rise in maternal BMI. After adjustment, the outcomes persisted in the groups with BMI 25–29.9 and 30–34.9. This result is consistent with the results of previous studies [10, 11, 13, 14, 16]. There were less women in G4 compared to the other BMI groups, which could explain why the result isn’t significant for these women,.

An increased incidence of VE was seen in normal-weight women with IOL compared to overweight and obese women. This has also been reported by Arrowsmith et al. in their 2011 study [11]. A significant increase in the risk for CS was seen in nulliparous women with increasing age and parity seems to be the strongest riskfactor for CS. The risk of CS after IOL increased significantly if the indication for IOL was maternal, as in the case of rising blood pressure, PE, pain, or other illness. One explanation could be that deterioration in the woman’s condition may have interrupted the delivery.

Why do women with a higher BMI undergo CS to a greater extent after IOL than normal-weight women? As indicated in previous studies, with a higher BMI, the risk of prolonged labor progress of up to six centimeters was seen during labor. Although cervical ripening is often slower than in normal-weight women, studies have shown that the latter part of the delivery is as fast as in normal-weight women [10, 13, 17]. However, this is often interpreted as primary dystocia and is associated with an increased risk of unnecessary interventions, which increases the risk of CS. This could explain the results of this study that the higher proportion of CS differed significantly between the BMI groups. Time from induction to delivery were longer in the groups with higher BMI even though it was not significant (p = 0.06). Together with previous results and WHO guidelines, this study confirmed that the latency phase extends to a cervical dilatation of five centimeters [18]. It demonstrates the importance of knowledge about how cervical ripening and opening progress during active labor differ with varying BMI. Thus, it is essential to consider this in clinical practice to reduce the risk of CS, in order not to interrupt the labor when the woman has not yet entered the active phase. The results of the study also showed that there are higher proportions of women undergoing CS due to primary dystocia among overweight and obese women than among normal weight women. Although the proportion of primiparous women was higher among those with a BMI <25, the proportion of CS was lower, perhaps because the obese and overweight women have not reached active labor before it is interrupted.

There was no difference in PPH between the BMI groups. Results from other studies are variable, with some showing similar results [15, 19], while others showing increased risk of PPH among obese women [7]. The reason for this was not investigated in this study. However, some speculation can be made. In the labor of overweight and obese women, there is often a pronounced concern about heavy bleeding after delivery, and rapid measures are taken to prevent this potentially life-threatening scenario. The success of this could be due to a higher preparedness for major bleeding and one of the reasons why there was not an increased risk in the study.

Obese and overweight women are at increased risk of complications during pregnancy and labor, and fetal weight is higher at birth. This may lead to complications seen in children large for gestational age, such as prolonged labor, shoulder dystocia, and instrumental delivery (VE and CS). The children of women with a higher BMI were seen in the study to have a significantly increased risk of fetal weight over 4000 g; otherwise, no significant difference in fetal data was observed. These results are consistent with those of previous studies [5, 11].

### Strengths and limitations

One of the strengths of this study is that it was carried out in one of Sweden’s largest delivery wards, with data from several years. Thus, the results can be generalized to a similar context in other parts of Sweden and Europe. It was a retrospective study; however, given that careful journal reviews were conducted of the included women, the results are more reliable compared to other retrospective studies based on, for example, diagnostic codes. The distribution of women with overweight and obesity in the study is consistent with the distribution in Stockholm, which is a strength of the study, as it reflects the population. A limitation with this group of women in Stockholm with the lowest BMI in the country, does not reflect the whole country. Another strength of the study is that baseline data did not differ significantly between the groups in terms of gestational age and BS, maternal age was significant different but the difference was 33.1–33.9 years and that difference in less than one year is of no clinical relevance, which is why it cannot be an explanation for the differences observed in the results of the study. A limitation may be that the study was conducted in only one hospital; a multicenter study would have been better, as a larger population from several contexts provides a greater generalization. We also observed a difference regarding parity, with more primiparous women in the normal weight group (G1). This difference can also be seen as a strength, as there is an increased risk in nulliparous women, with delivery tending to end by CS. Further, the proportion of nulliparous women in the study was lower among the overweight and obese groups. The increased risk of CS observed in women with BMI>25 was not explained by parity.

## Conclusion

The findings of this study showed that a higher BMI increased the risk of CS after IOL in the groups G2 and G3 (BMI 25–34.9). Overweight and obese women were at increased risk of complications during pregnancy and labor. Parity seems to be the strongest risk factor for CS regardless other variables. The largest proportion of primiparous women was seen in the group with lowest BMI that also had the fewest CS. Thus, it is important to consider these findings to increase the chance of vaginal labor, avoid an unnecessarily CS, and reduce the risks of future pregnancy and labor.

## Supporting information

S1 Data (XLSX)

## Abbreviations

BMI: Body mass index (weight in kg/length m2)
BS: Bishop score
CS: cesarean section
CI: confidence interval
GI: women with BMI ≤24.9
G2: women with BMI 25–29.9
G3: women with BMI 30–34.9
G4: women with BMI ≥35
IUFD: intrauterine fetal death
LGA: large for gestational age
OR: odds ratio
PE: preeclampsia
VE: vacuum extraction

## References

[1] Förekomst av övervikt och fetma Folkälsomyndigheten.se: Folkhälsomyndigheten; 2020 [updated 2020-10-27]. Available from: https://www.folkhalsomyndigheten.se/livsvillkor-levnadsvanor/fysisk-aktivitet-och-matvanor/overvikt-och-fetma/forekomst-av-overvikt-och-fetma/.

[2] Bjermo H, Lind S, Rasmussen F. The educational gradient of obesity increases among Swedish pregnant women: a register-based study. BMC Public Health. 2015;15:315. Epub 2015/04/18. doi: 10.1186/s12889-015-1624-6 ; PubMed Central PMCID: PMC4391086. 25886465 PMC4391086

[3] Statistik om graviditeter, förlossningar och nyfödda barn 2017 Socialstyrelsen.se [updated 2019-05-02]. Available from: https://www.socialstyrelsen.se/globalassets/sharepoint-dokument/artikelkatalog/statistik/2019-5-2.pdf.

[4] Åmark H. Pregnancies Complicated by Obesity- Focus on Stillbirth and Infants Born Large for Gestational Age [doctoral degree]: Karolinska institutet; 2020.

[5] Kim SS, Zhu Y, Grantz KL, Hinkle SN, Chen Z, Wallace ME, et al. Obstetric and Neonatal Risks Among Obese Women Without Chronic Disease. Obstetrics and gynecology. 2016;128(1):104–12. Epub 2016/06/09. doi: 10.1097/AOG.0000000000001465 ; PubMed Central PMCID: PMC4917420. 27275800 PMC4917420

[6] Wolfe KB, Rossi RA, Warshak CR. The effect of maternal obesity on the rate of failed induction of labor. American journal of obstetrics and gynecology. 2011;205(2):128.e1–7. Epub 2011/05/31. doi: 10.1016/j.ajog.2011.03.051 . 21621187

[7] Lassiter JR, Holliday N, Lewis DF, Mulekar M, Abshire J, Brocato B. Induction of labor with an unfavorable cervix: how does BMI affect success? (‡). The journal of maternal-fetal & neonatal medicine: the official journal of the European Association of Perinatal Medicine, the Federation of Asia and Oceania Perinatal Societies, the International Society of Perinatal Obstet. 2016;29(18):3000–2. Epub 2015/10/30. doi: 10.3109/14767058.2015.1112371 . 26513375

[8] Dammer U, Bogner R, Weiss C, Faschingbauer F, Pretscher J, Beckmann MW, et al. Influence of body mass index on induction of labor: A historical cohort study. J Obstet Gynaecol Res. 2018;44(4):697–707. Epub 2018/01/10. doi: 10.1111/jog.13561 . 29316054

[9] Rogers AJG, Harper LM, Mari G. A conceptual framework for the impact of obesity on risk of cesarean delivery. American journal of obstetrics and gynecology. 2018;219(4):356–63. Epub 2018/06/15. doi: 10.1016/j.ajog.2018.06.006 . 29902446

[10] Ellis JA, Brown CM, Barger B, Carlson NS. Influence of Maternal Obesity on Labor Induction: A Systematic Review and Meta-Analysis. Journal of midwifery & women’s health. 2019;64(1):55–67. Epub 2019/01/17. doi: 10.1111/jmwh.12935 ; PubMed Central PMCID: PMC6758543. 30648804 PMC6758543

[11] Arrowsmith S, Wray S, Quenby S. Maternal obesity and labour complications following induction of labour in prolonged pregnancy. BJOG: an international journal of obstetrics and gynaecology. 2011;118(5):578–88. Epub 2011/01/27. doi: 10.1111/j.1471-0528.2010.02889.x ; PubMed Central PMCID: PMC3085126. 21265999 PMC3085126

[12] Stephansson O PK, Bjork C, Conner P, Wikstrom AK. The Swedish Pregnancy Register–for quality of care improvement and research. Acta Obstet Gynecol Scand. 2017. 10.1111/aogs.13266 PMC5873375 29172245

[13] Carpenter JR. Intrapartum Management of the Obese Gravida. Clin Obstet Gynecol. 2016;59(1):172–9. Epub 2016/01/13. doi: 10.1097/GRF.0000000000000174 . 26756259

[14] Vinturache A, Moledina N, McDonald S, Slater D, Tough S. Pre-pregnancy Body Mass Index (BMI) and delivery outcomes in a Canadian population. BMC Pregnancy Childbirth. 2014;14:422. Epub 2014/12/22. doi: 10.1186/s12884-014-0422-y ; PubMed Central PMCID: PMC4300169. 25528667 PMC4300169

[15] Kobayashi N, Lim BH. Induction of labour and intrapartum care in obese women. Best practice & research Clinical obstetrics & gynaecology. 2015;29(3):394–405. Epub 2014/12/03. doi: 10.1016/j.bpobgyn.2014.07.024 . 25441151

[16] Tolcher MC, Holbert MR, Weaver AL, McGree ME, Olson JE, El-Nashar SA, et al. Predicting Cesarean Delivery After Induction of Labor Among Nulliparous Women at Term. Obstetrics and gynecology. 2015;126(5):1059–68. Epub 2015/10/08. doi: 10.1097/AOG.0000000000001083 ; PubMed Central PMCID: PMC4618703. 26444107 PMC4618703

[17] Harper LM, Caughey AB, Odibo AO, Roehl KA, Zhao Q, Cahill AG. Normal progress of induced labor. Obstetrics and gynecology. 2012;119(6):1113–8. Epub 2012/05/10. doi: 10.1097/AOG.0b013e318253d7aa . 22569121

[18] WHO Guidelines Approved by the Guidelines Review Committee. WHO recommendations: Intrapartum care for a positive childbirth experience. Geneva: World Health Organization Copyright © World Health Organization 2018.; 2018. 30070803

[19] Athukorala C, Rumbold AR, Willson KJ, Crowther CA. The risk of adverse pregnancy outcomes in women who are overweight or obese. BMC Pregnancy Childbirth. 2010;10:56. Epub 2010/09/21. doi: 10.1186/1471-2393-10-56 ; PubMed Central PMCID: PMC2949787. 20849609 PMC2949787

